# Role of Regulatory T Cells in Disturbed Immune Homeostasis in Patients With Chronic Obstructive Pulmonary Disease

**DOI:** 10.3389/fimmu.2020.00723

**Published:** 2020-04-28

**Authors:** Jia Hou, Yongchang Sun

**Affiliations:** ^1^Department of Respiratory and Critical Care Medicine, General Hospital of Ningxia Medical University, Ningxia, China; ^2^Department of Respiratory and Critical Care Medicine, Peking University Third Hospital, Beijing, China

**Keywords:** COPD, T-regulatory cells, immune homeostasis, adaptive immunity, smoking, aging

## Abstract

Chronic obstructive pulmonary disease (COPD) is a complex chronic disease in which T cell-mediated pulmonary inflammation has been shown to play a key role. Accumulating evidence shows that COPD has many of the characteristics of an autoimmune response. An adaptive immune response directed against lung self-antigens, which are released during the initial innate inflammatory response and are triggered by constant exposure to cigarette smoke and epithelial injury, drives the persistent inflammatory response found in smokers. The development and severity of adaptive inflammation depend on the level of tolerance to self-antigens. For these reasons, the effect of regulatory T (Treg) cells on adaptive immunity in COPD patients is of particular interest and could be targeted therapeutically. The disturbance in immune homeostasis caused by changes in the number or function of Treg cells, which is related to cigarette smoke exposure, may be of importance in understanding the development and progression of COPD.

## Background

Chronic obstructive pulmonary disease (COPD) presents as a persistent limitation in airflow that usually becomes progressively worse and is associated with exacerbated inflammatory responses of the lungs to irritating gases or particles (1). Cigarette smoke is the primary cause of COPD, yet inflammation persists even after individuals cease smoking. Accumulating evidence (2–5) shows that adaptive immunity directed against lung self-antigens plays important roles in perpetuating the immune response, well after the initial trigger of cigarette smoke is no longer present. The immunoregulatory capacity of this adaptive inflammation determines the susceptibility to, and severity of, disease. The precise regulatory mechanisms in COPD, however, remain poorly understood. Among several distinct subsets of T cells with regulatory functions, regulatory T (Treg) cells expressing the transcripting factor Foxp3 are a subpopulation of CD4^+^ lymphocytes that have a major effect on autoimmunity. The question arises whether quantitative and/or functional defects of Treg cells are linked to the development of COPD.

In the present review, we will briefly summarize both current understanding of the functions of Treg cells and evidence for their involvement in the pathogenesis of COPD.

## Development of Treg Cells

Since the regulatory T cells was first discovered in 1969 (6), this field of research has undergone an incredible boom over the past 50 years. Recent advances in the biology of Tregs are now providing unprecedented levels of information pertaining to the development and differentiation of Tregs. The thymus is a crucial organ for the generation and development of Tregs. It has long been proposed that progenitors of Treg cells originate in the bone marrow, then mature in the thymus upon recognition of thymic epithelial or dendritic cell self-antigens (7). Immature thymocytes, however, also show an inclination toward Treg differentiation in the thymus by an antigen-presenting cell (APC)-free intrinsic control mechanism, that only requires T cell receptor (TCR) stimulation and IL-2 signaling (8). TCR stimulation is crucial for differentiation of Tregs in the thymus. During thymic development, most immature T cells are eliminated via negative selection following the TCR signal, whereas a minority differentiate into thymus-derived Tregs (tTregs). Apart from TCR signals, CD28 is also indispensable for the production of tTregs. It has been demonstrated that CD28 co-stimulation can significantly induce thymocytes to express Foxp3 independently of IL-2. CD28 deficiency in mice result in decreased number of tTreg (9). In addition to tTregs produced in the thymus, peripheral naïve T cells show upregulated Foxp3 expression and acquire Treg phenotypes and functions following treatment with TGF-β *in vitro* or chronic antigen stimulation *in vivo*, particularly at sites of inflammation and environmental interfaces, such as the gut (10–12). Not surprisingly, these cells are generally assumed to play a major role in maintaining mucosal immune tolerance. Such induced Treg (iTreg) cells are, however, functionally unstable because they lack the full demethylation that underpins the Treg signature genes. The extent to which induced Foxp3^+^ Tregs contribute to systemic self-tolerance remains unclear and there is also no consensus about which cell markers can be used to distinguish tTreg and iTreg cells and thus allow the properties of each type of cell to be studied separately (13).

Foxp3, a forkhead family transcription factor, is essential for the development and suppressive function of Treg cells. Mice with the *Scurfy* mutation in the *Foxp3* gene show lymphoproliferation and autoimmune responses in multiple organs and similar symptoms are seen in humans with mutations in *Foxp3* who develop a severe, systemic autoimmune disorder called immune dysregulation polyendocrinopathy enteropathy, X-linked (IPEX) syndrome (14). Foxp3 can regulate a large number of gene expressions during differentiation by binding to >2800 genetic loci in precursor and mature Tregs (15, 16). Although Treg cell-specific transcription factors are induced by TCR stimulation and IL-2 signaling, an appropriate chromatin structure is also necessary for transcription factors to selectively combine with their target sequences. It has been shown that DNA demethylation status of Treg signature genes is essential for maintaining stable human Tregs lineage. Specifically, CNS2 element within the first intron of Foxp3 gene (also known as T reg cell–specific demethylated region, TSDR) is critical for maintenance of Foxp3 in tTreg. In contrast, iTreg cells generally exhibit a methylated or partially demethylated CNS2 element, and are considered functionally unstable. Together, these findings suggest that epigenetic regulation is also crucial to initiate Treg cell development and to maintain stable suppressive function at the genomic level.

In addition, intracellular metabolic changes are also important factors modifying the development and suppressive function of Tregs. Previous research has shown that T helper (th) cell rely primarily on glycolytic metabolic pathways for proliferation. In contrast, established Treg cells rely more on mitochondrial oxidation pathways for their suppressive function. Both pathways actually represent two metabolism modulating mode. Glycolytic metabolism allows inflammation, whereas oxidative metabolism suppress inflammation. It has been shown that inhibiting glycolysis results in increased expression of Foxp3 during iTregs development (15, 17). Those findings suggest that metabolism modulate the functional balance between proliferation and suppression in Tregs. Gerriets et al. (18) found signaling via the Toll-like receptor (TLR) activated PI3K-AKT-mTORC1 axis in Tregs, and this pathway promotes proliferation of Tregs by supporting glycolysis. The suppressive function of Tregs, however, was also impaired at the same time. Conversely, Foxp3 is capable of affecting metabolism in Tregs by modulating the genes that encode PI3K subunit. Together, Foxp3 formed a strong interaction with intracellular metabolism in the differentiation of Tregs.

## Mechanisms of Treg-Cell Function

Treg cells provide essential protection to the body against an overactivated immune response. Reduced numbers and/or functional impairment of Treg cells is found in a number of immune-related diseases (19). It is, therefore, important to understand Treg cell-mediated immunosuppressive mechanisms. This may not only provide insights into disease pathogenesis but could also provide a number of potentially important therapeutic targets.

Treg cells most likely exert their suppressive effects by multiple mechanisms. It has been reported that activated human Treg cells kill effector cells or APCs by releasing granzyme A and perforin (20), or functionally modulate them via CTLA-4 and CD80 and/or CD86, which are co-stimulatory molecules expressed on dendritic cells (21, 22). Other mechanisms are mediated by soluble factors. For example, it has been shown that the immunosuppressive molecules IL-35, IL-10, TGF-β, and LAG3 are critical mediators of Treg function (23–25). ICOS^+^Foxp3^+^ Treg cells can suppress dendritic cell and T cell functions through IL-10 and TGF-β, respectively (26), whereas HLA-DR^+^ Treg cells induce early contact-dependent suppression (27). Treg cells also use many other inhibitory molecules, such as CD39, CD73 and T-cell immunoglobulin and ITIM domains (TIGIT), to suppress the immune response (28). Human Tregs are also known to cause senescence in responder effector and naïve T cells, both *in vitro* and *in vivo*. Responder T cells with induced senescence have altered cytokine profiles and phenotypes and are potently suppressive. Treg-mediated control of responder T cell senescence is associated with selective regulation of ERK1/2 signaling, p38, and cell cycle regulatory factors p21, p16, and p53v, adding novel mechanisms for human Treg cell suppression (29).

Another key target for Tregs is dentritic cells (DCs). More recently, the mechanisms of action of Tregs have been expanded by several studies on their interactions with dendritic cells. Akkaya et al. (30). demonstrated that antigen specific Tregs downregulate antigen presenting function of DCs by depleting peptide-MHCII from the DCs surface. Notably, this process is antigen specific and represent a novel mechanism for Tregs suppressive function. Another important pathway by which Tregs modulate DCs function is via the transfer of miRNA from Tregs to DCs, in particular miR-150-5p and miR-142-3p (31). Both miR-150-5p and miR-142-3p are thought to upregulate IL-10 and downregulate IL-6 productions (32).

In addition to classical immunosuppressive functions, several studies have revealed much broader and multilevel mechanisms by which Treg cells regulate immunity and maintain tissue homeostasis. For example, recruitment of Treg cells to injured skeletal muscle to promote tissue repair was found to be mediated by the growth factor amphiregulin and regulated by IL-18 and IL-33 (33). Deficiency in amphiregulin results in severe acute pulmonary injury during influenza virus infection, without any changes in the capacity of the Treg cell for immunosuppression. Such tissue repair modalities are not driven by TCR signaling, which is indispensable for the immunosuppressive effects of Treg cells (34), suggesting that these two distinct functions of Treg cells are invoked by separate pathways. These observations are noteworthy since they may provide further insights into the role of Treg cells in disease.

## Phenotypic Characteristics of Treg Cells

Identifying specific markers that define Treg cells and distinguish them from activated effector T cells is important to completely understand the function of these cells. There is, however, still no consensus on reliable specific markers for identifying Treg cells.

The transcription factor Foxp3 has long been considered to be essential for the function and development of CD4^+^CD25^+^ Tregs (35, 36). It has been found that T cells can express the *Foxp3* gene in specific situations. The cells then acquire immunosuppressive activity, indicating that Foxp3 expression plays a regulatory role (37). Foxp3 is, therefore, deemed to be the most accurate intracellular marker of Treg cell activity identified so far. It has, however, been suggested that Foxp3 is not a bona fide marker of tTregs since Foxp3 is transiently upregulated in effector T cells upon activation (38) and Treg cells can also lose Foxp3 expression and convert to effector T cells (39). Similarly, a variety of specific Treg markers, such as glucocorticoid-induced tumor necrosis factor receptor, CD25, adhesion molecule CD62L, PD-1, cytotoxic T lymphocyte antigen-4 (CTLA-4) and Helios, are also upregulated upon activation (40, 41). CD127, a surface marker used to isolate genuine human Treg cells via flow cytometry, is not a specific marker (42). A possible mechanism underlying this phenomenon is the disparity in CpG methylation levels at Treg-exclusive genes. DNA demethylation is indispensable for the stable phenotype and function of Treg cells (43).

Whether there is an exclusive marker, expressed only by tTreg cells, has been questioned. The discovery of a transmembrane protein called GARP (glycoprotein A repetitions predominant, or LRRC32) represents an advance in this area (44). Tregs exert immuno-regulatory function by converting the latent TGF-β1 presented by GARP into active TGF-β1, which is highly immunosuppressive. This whole process requires the presence of integrin αVβ8. Blocking anti-GARP or anti-β8 mAbs is potentially a novel immunotherapeutic approach to dampen Treg function (45, 46). Interestingly, human B lymphocytes also secret TGF-β1 upon stimulation in a GARP dependent manner (47, 48). Clarifying this mechanism is helpful to predict potential adverse effects of anti-GARP mAbs therapy. Expression of GARP has been reported to occur only in activated human tTregs and their clones, and not in activated effector T cells, indicating that GARP is a true tTreg cell marker (49, 50).

## Division of Treg Cells Into Three Functionally Different Subsets

Over the years, people have gradually realized that Treg cells are a heterogeneous cell population. This is a milestone in optimizing the efficacy of human Treg immunotherapy by selecting functionally different subpopulations of Tregs and identifying the ideal source of Tregs. Thus far, > 40 Treg subpopulation divisions defined by different surface molecules have been reported (51, 52).

The most widely accepted classification method was reported by Miyara et al., which divides human Treg cells into three functionally and phenotypically separate subpopulations (53). These are: CD45RA^+^Foxp3^low^ resting cells (rTregs, fraction I) and CD45RA^−^Foxp3^hi^ activated cells (aTregs, fraction II), both of which are suppressive *in vitro*, and pro-inflammatory cytokine-secreting CD45RA^−^Foxp3^l^° cells (fraction III), which are non-suppressive. CD45RA^+^ rTreg cells are bona fide suppressive Treg cells in which the TSDR within the Foxp3 enhancer is demethylated. This subpopulation predominates in cord blood and decreases with age because of thymic involution. Once activated, rTreg cells enhance Foxp3 expression, then actively proliferate, and finally convert to aTreg cells. As the main effectors of suppression, aTreg cells have the highest expression of Foxp3 and CD25 among the three subpopulations of Treg cells, show high capacity for immunosuppression and have a demethylated TSDR. Interestingly, rTreg and aTreg cells use different inhibitory mechanisms, associated with production of different anti-inflammatory cytokines, including IL-10 and TGF-β. In contrast, cells in fraction III shows low levels of TSDR demethylation, secrete pro-inflammatory cytokines and show decreased suppressive activity. More importantly, these cells express both Foxp3 and RORγt, have the potential to differentiate into Th17 cells and produce IL-17, IL-2, and TNF-α under inflammatory conditions (53). Several studies have suggested that these Foxp3-RORγt double-positive CD4^+^ T cells can be converted into either Tregs or Th17 cells, depending on the cellular micro-environment (54, 55), and that immature granulocytes or myeloid-related chemokines induce the conversion of pro-inflammatory cytokine-secreting Tregs into aTreg cells (56). Therefore, the capacity of producing pro-inflammatory cytokines was thought to be an early indicator of weakening lineage fidelity. The ability of the cells to retain their suppressive function, however, remains controversial (53, 57, 58). IL-17A-producing CD4^+^Foxp3^+^ Treg cells are known to be involved in various diseases, including COPD and asthma (59–61).

The proportions of these Treg subpopulations differ between cord blood, the elderly and patients with immunological diseases (53). Examining Foxp3^+^ cell subsets allows analysis of Treg cell differentiation and of differences between normal and diseased states (56, 62–64). Manipulating these subpopulations may also enable therapeutic control of the immune response.

Of all the division methods, the most basic subdivision separates human Tregs into naïve or memory subsets based on the expression of CD45RA or CD45RO (51). In terms of gene expression profiles, naïve Tregs and memory Tregs exhibit different biological features and represent distinct subsets. The HLA-DR^+^ memory Tregs (DR^+^ mTreg) have potent immuno-suppressive function with limited proliferative capacity, whereas HLA-DR^−^ memory Tregs (DR–mTreg) are less suppressive, but more proliferative. In contrast to mTreg subpopulations, naïve Tregs exhibit the least suppressive capacity, but the greatest proliferative capacity (27). Naïve Tregs, however, become more suppressive upon activation. The *in vitro* test further demonstrated that both DR^+^ mTreg and DR^−^ mTreg are not suitable for a therapeutic approach due to poor lineage stability, limited proliferation capacity and increased propensity to differentiate into pro-inflammatory cells. In comparison, naïve Tregs possess all the ideal properties needed for Treg therapy, such as potent suppressive capacity, strong lineage fidelity and considerable expansion *in vitro* (52). Collectively, those findings offer a promising roadmap for optimizing human Treg therapeutics.

## The impact of aging on Treg cells

COPD has long been regarded as an age-related disease. The incidence of COPD is much higher in people over the age of 60 years than in younger age groups (65, 66). The mechanism underlying the greater susceptibility of elderly people to COPD remains poorly understood. COPD is characterized as a chronic inflammatory disorder, involving both the innate and adaptive immune systems. The effect of aging on the immune system, termed immunosenescence, has also been shown to involve both innate and adaptive immune systems (67) and, unsurprisingly, immunosenescence is considered to be a critical contributor to the development of COPD.

Changes in T cell immunity, including prevalence and activities of subsets of T cells, occur with aging. Senescence of T lymphocytes might contribute to the pathogenicity of a variety of chronic diseases of the elderly, including cancer, autoimmune diseases, neurological disorders, and COPD. (68, 69) Progressive degeneration of the thymus occurs during aging and results in loss of its ability to produce and renew T cells, including tTregs (69, 70). The total prevalence of Tregs in the periphery, however, remains unchanged because enhanced peripheral generation of Tregs compensates for progressive thymic degeneration (69, 70). Consequently, numbers of naïve Tregs decrease, whereas those of memory Tregs and iTregs increase, during aging (71). TCR diversity is also significantly reduced in elderly individuals (72). The alterations in Tregs during aging lead to declines in the adaptive immune response and an increased risk of immune-mediated disorders (73). The accumulation of Treg cells in old age helps to explain some of the immunological diseases typical of aging, including malignancies, infections, and COPD. In addition to impairing adaptive immune responses, aging also affects the innate immune system and leads to chronically elevated basal levels of systemic inflammation, characterized by elevated pro-inflammatory cytokines, such as IL-1, IL-6, and TNF-α. This phenomenon has been termed “inflammaging” (67) and, interestingly, this senescence-induced pro-inflammatory state is similar to that observed in COPD (65).

Given the physiological and immunological similarities between COPD and aging, COPD was regarded to be an “accelerated aging phenotype.” Aging and COPD are mediated by common molecular mechanisms, including increased activation of NF-κB, oxidative stress, telomere shortening and impaired DNA repair, leading to significant dysregulation of the immune system (74). Evidence suggests that accelerated aging, which can be caused by cigarette smoking in susceptible individuals, is a risk factor for COPD. Immunosenescence may, therefore, be a driving mechanism in COPD and regulation of immuno-senescence could be a promising way to slow down the development of COPD.

## Role of Tregs in Disturbed Immune Homeostasis in COPD

Evidence suggests that autoimmunity is important in the pathogenesis of COPD (2, 75). During the immune response, Treg cells act as a negative regulatory population. In theory, Treg cells could either offer protection against, or induce susceptibility to, autoimmune diseases. Their precise role in the pathological mechanisms underlying COPD, however, remain unclear and previous studies in humans have revealed discrepant results.

As compared with never-smokers, bronchoalveolar lavage (BAL) fluids from smokers with normal lung function have markedly upregulated CD4^+^CD25^+^ T cells, which are absent in COPD patients (76). Similar results were found by Roos et al., who found that CD4^+^CD25^+^ cells, with little or no CD127 expression, are more prevalent in smokers with typical lung function compared with nonsmokers, but not compared with COPD patients (77). In line with these results, Chu et al. found fewer Foxp3-positive cells in the alveolar compartments of COPD patients compared with smokers and control groups (78). Smokers with COPD also have significantly fewer Treg cells and less Foxp3 mRNA in the lungs than healthy smokers (79) and Chiappori et al. demonstrated that a lower number of circulating Treg cells is associated with a greater decline in FEV_1_ (forced expiratory volume in the first second) (80). In contrast, increased proportions of CD4CD25(bright) have been found in BAL fluid from smokers with COPD, compared with controls (81). Increased numbers of CD4CD25(bright) cells in the lungs are, however, more related to long-term exposure to cigarette smoke than to development of airflow obstruction (81), suggesting that long-term cigarette smoking increases Treg cells in the airways. However, expression of FoxP3 was confirmed in only four subjects, which diminished the value of this research.

In COPD patients, the proportion of Treg cells fluctuates, not only in BAL fluid but also in various pulmonary tissues. Higher numbers of CD4^+^Foxp3^+^ T cells have been found in pulmonary lymphocyte follicles of moderate COPD patients, but not in lung parenchyma (82). Another study found that Foxp3-positive cells are upregulated in large airways but downregulated in small airways, the main site of pathological involvement in COPD, which correlates with limited airflow (83). Similarly, Sales et al. found decreased density of Treg, TGF-β^+^ and IL-10^+^ cells in small airways only in COPD individuals, which resulted in modified and persistent inflammation in lung tissue (84). These studies imply that the role of Treg cells in regulation of the immune response may vary in different lung regions and this requires further clarification. More recently, it has been shown that CD4^+^CD25^−^Foxp3^+^ T cells, which have the potential to differentiate into pro-inflammatory Th17 cells, might perpetuate chronic inflammation in COPD (85). Interestingly, the percentage of CD4^+^CD25^−^Foxp3^+^ T cells possibly correlates with CD4^+^CD25^+^Foxp3^+^ T cells in acute exacerbations of COPD, but not in stable COPD, suggesting immune homeostasis between these cells in the different phases of the disease.

In our study, we observed increased frequencies of both inhibitory (including aTreg and rTreg cells) and pro-inflammatory subpopulations in smokers, suggesting that inflammatory and anti-inflammatory responses coexist. Compared with healthy smokers, COPD patients show a higher proportion of pro-inflammatory subpopulations and a lower proportion of inhibitory subpopulations. This could reflect the progression of inflammation and the exhaustion of anti-inflammatory cells during the development of COPD (61).

**Figure 1:**
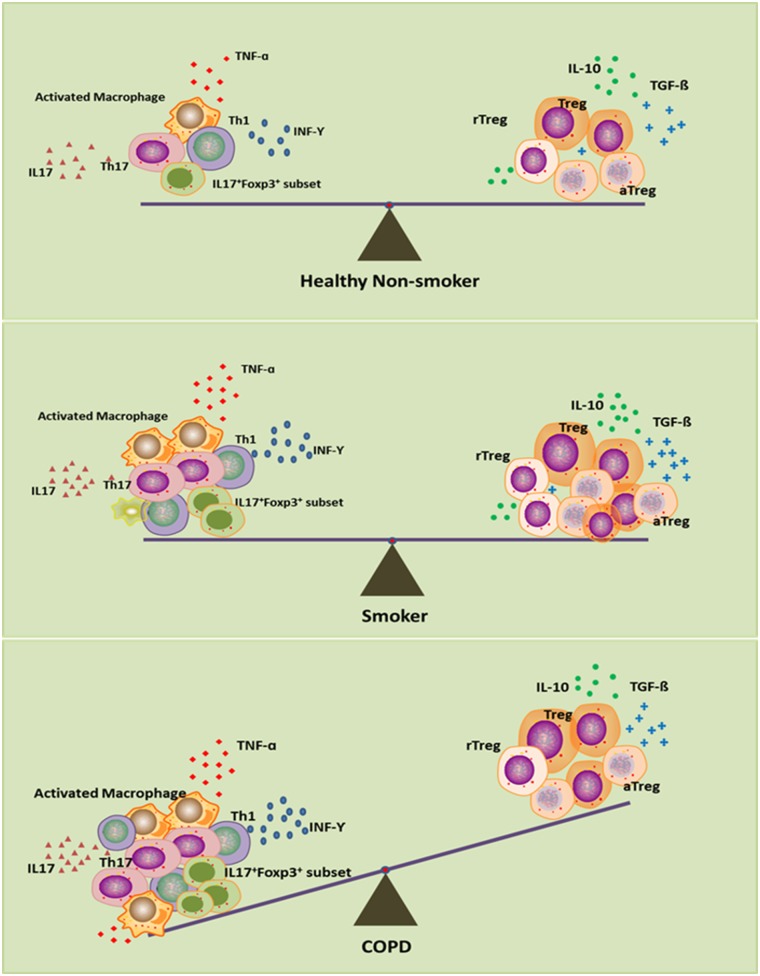
Disturbed immune homeostasis in patients with chronic obstructive pulmonary disease (COPD). In smokers, although smoke exposure triggers the pro-inflammatory response, the compensatory anti-inflammatory mechanism keeps immune homeostasis intact as in healthy non-smokers. In smoking COPD patients, long-term smoking can lead to exhaustion of the compensatory anti-inflammatory compacity, which leads to the imbalance between pro- and anti-inflammatory mechanisms, and eventually disrupts the immune homeostasis.

Overall, the picture that emerges from all these data is that Treg cells in smokers with normal lung function may be increased in an attempt to control pulmonary inflammation, maintain local immune homeostasis and eventually prevent the development of COPD. Those smokers without COPD or with mild COPD thus have reduced inflammation (86–88). Impairment of Treg cells might destroy this homeostasis and predispose to perpetual pulmonary inflammation, with severe lung damage and COPD. This immune homeostasis exists not only between Treg cells and inflammatory cells, including Th17, Th1, and CD8^+^ T cells, but also within different functional subsets of Treg cells (Figure 1). This hypothesis may help to explain why lung cancer occurs more frequently in smokers with mild airflow limitation (GOLD Stages I and II) than in smokers with severe COPD (89) and why more autoimmune diseases are associated with severe COPD than with lung cancer (90).

## Treg Cells in COPD: More Than a Numbers Game

Treg cells play a critical role in the suppression of inflammatory pathology. It has been shown that rescue of Treg number and/or function can both prevent and reverse disease. The treatment for the restoration of disturbed immune homeostasis is dependent upon accurate evaluating of the frequency and/or function of Tregs. Most studies have identified Treg cells by surface markers and/or Foxp3 expression (Table 1). However, all of these markers, including Foxp3, are also expressed on activated effector T cells, particularly at sites of inflammation. Activated T cells may thus have been identified as Treg cells in these studies, underscoring the need for functional assays to evaluate the role of Tregs in COPD. Evaluating overall defects in Treg cells in COPD patients has, however, been challenging.

**Table 1:** Expression of data in COPD.

**References**	**Patients**	**Compartment**	**Defining method**	**Functional assay**	**Analysis**	**Key findings**
Barcelo et al. (58)	Never-smokers 7 Smokers 29 COPD 23	BALF and PB	CD4^+^CD25^+^	No	FACS	No differences in PB Decrease in BALF of COPD compare to smokers
Roos et al. (59)	Never-smokers 9 Smokers 14 COPD 9	BALF and PB	CD4^+^CD25^bright^CD127^−^	No	FACS	Increased BAL Tregs in smokers
Chu et al. (60)	Never-smokers 10 Smokers 10 COPD 10	Lung tissue	CD4^+^Foxp3^+^	No	Immuno-histochemistry	Decreased Foxp3^+^ in COPD
Lee et al. (61)	Never-smokers 7 Emphysema 14	PB and lung tissue	CD4^+^CD25^hi^CD62L^+^	No	FACS	Decreased Tregs in lungs of emphysema
Chiappori et al. (62)	Healthy controls 20 COPD 28	PB	CD4^+^CD25^+^CD127^low^	No	FACS	Reduced Tregs in COPD
Smyth et al. (63)	Never-smokers 8 Smokers 19 COPD 26	BALF and PB	CD4+CD25bright	No	FACS	Increased BAL Tregs in smokers and COPD
Plumb et al. (64)	Never-smokers 7 Smokers 11 COPD 12	Lung tissue	CD4+Foxp3+	Yes	Immuno-histochemistry	Increased Tregs in Lymphoid follicles of COPD patients
Isajevs S et al. (27)	Never-smokers 19 Smokers 20 COPD 20	Lung tissue	Foxp3^+^	No	Immuno-histochemistry	Upregulation in large airways, downregulation in small airways
Sales et al. (65)	Healthy controls 21 Smokers 22 AECOPD 13	Lung Tissue	Foxp3^+^, TGF-ß^+^, IL-10^+^	No	Immuno-histochemistry	Decreased Treg in small airways of COPD
Hou et al. (40)	Never-smokers 57 Smokers 32 COPD 66	PB and BALF	aTreg:CD4^+^Foxp3^+^CD45RA^−^ rTreg:CD4^+^Foxp3^+^CD45RA^+^ FrIII : CD4^+^Foxp3^+^CD45RA^−^	Yes	FACS	Decreased rTreg, aTreg and increased FrIII in COPD
Tan et al. (66)	Healthy controls 15 COPD 14	PB	CD4^+^CD25^+^CD127^low^	Yes	FACS	Impaired function of Tregs in COPD
Kalathil et al. (69)	Healthy controls 31 COPD 24	PB	CD25^+^CD127^−^Foxp3^+^	No	FACS	Increased Tregs number and suppressive function
Tan et al. (70)	Healthy controls 26 Stable COPD 24 AECOPD 17	PB	CD4^+^CD25^+^Foxp3^+^CTLA^+^	No	FACS	Increased in AECOPD

Lee et al. (79) carried out functional assays using Tregs from emphysema patients and healthy controls. Circulating Tregs from both groups had markedly inhibited autologous T cells proliferation, whereas IL-10 secretion from the whole lung of emphysema patients was reduced compared with control subjects, suggesting normal peripheral Treg capacity but impaired pulmonary Treg function in emphysema patients. More recently, a subset of COPD patients with high body mass index was found to have similar proportions of circulating Treg cells compared to controls, but impaired suppression of CD4^+^ T-cell activation by Tregs (91). The take-home message from this study is that heterogeneity of disease might underlie the observed discrepancies in Treg numbers and function. The role of Tregs may differ in different clinical phenotypes of COPD. It is also interesting that many CD4^+^CD25^+^ cells in BAL fluid of smokers do not express Foxp3 and therefore do not have regulatory T cell function (77). Another study also shows that markedly reduced lung CD4^+^ T cells polarization to T helper cells, including Tregs and aberrant inflammatory cytokines production following TCR stimulation, are associated with airflow limitation in COPD, implying a local immune deficiency and disorder involved in the mechanism underlying COPD (92). Collectively, these data suggest that a functional assay is essential to evaluate the role played by Tregs in COPD, and that more specific biomarkers are needed.

Thus far, there is little information about the suppressive ability of pulmonary Treg cells from COPD patients and most *in vitro* functional assays were performed using circulating Tregs. However, increasing evidence indicate that phenotype and frequency of Tregs in the peripheral blood and tissues are substantially different (93). The inflammatory milieu affects activation and function of Tregs in local tissues. For instance, a large number of memory T cells accumulate in human lungs to enable timely response to antigenic stimuli from the environment (94). Hence the discovery of circulating Tregs cannot be inferred to the phenotype, frequency and function of tissue Tregs. In summary, the study of pulmonary Treg cells in COPD is still in its infancy, there is an urgent need to determine the phenotype and function of pulmonary Treg cells in COPD with more innovative approaches.

## Treg and Corticosteroid Insensitivity in COPD

Although the majority of asthma patients can be well controlled with regular doses of corticosteroids, the majority of patients with COPD show reduced responsiveness to even high doses of local or systemic steroids. Evidence from *in vitro* studies also suggests that inflammation in COPD is not sensitive to corticosteroids (80), implying that steroid resistance is part of the underlying inflammatory mechanism. Poor understanding of the mechanism of steroid resistance is a major barrier to the effective treatment of COPD.

Corticosteroids have been shown to upregulate Foxp3 expression in asthma patients, in an attempt to restore the balance between effector T cells and Treg cells (95, 96). In some cross sectional studies, however, the number of Tregs in BAL fluid, or in the systemic circulation, of COPD patients was not affected by the use of corticosteroids (97), suggesting that regulatory T cells in these patients are insensitive to corticosteroids. An *in vitro* study confirmed that CD4^+^CD25^high^CD127^−^ cells from healthy controls and smokers, but not COPD patients, were significantly modulated by corticosteroids. The different behavior of Treg cells from COPD patients in *in vitro* studies suggests that immunological mechanisms may play important roles in corticosteroid resistance. Studies describing the effects of corticosteroids on regulatory T-cells in COPD patients are, however, sparse and sometimes conflicting. A prospective study is thus still needed to clarify the effects of corticosteroids on Treg cells.

Histone deacetylase (HDAC) family members play crucial roles in maintaining the lineage integrity of T cells, including Tregs (98). Since progressive reduction in HDAC activity, which is secondary to increased oxidative stress, reflects the severity of disease and accounts for corticosteroid resistance in COPD, it is reasonable to speculate that fluctuations of Tregs and the blunted response to corticosteroids are associated with changes in HDAC expression and activity. Further experiments are required to clarify the immunological mechanism of corticosteroid resistance in COPD.

## Tregs Can Be a Double-Edged Sword in COPD

Contrary to our understanding that a deficiency of Treg cells may predispose to autoimmunity and inflammation (99), Treg cells can also be considered to be promising therapeutic targets to diminish pulmonary inflammation and fibrosis in COPD and idiopathic pulmonary fibrosis (100). Several studies have confronted us with a different paradigm. Patients with COPD exhibit elevated levels of peripheral immunosuppressive cells, including Treg and myeloid-derived suppressor cells, together with increased numbers of exhausted T effector cells (programmed death 1 [PD-1]^+^), which appear to lack the energy to proliferate when exposed to bacteria (101). Chronic inflammation may expand subpopulations of T cells expressing CTLA-4 and Treg cells in COPD patients and therefore impair Th1 function (102). Both PD-1 blockade in T cells and CTLA-4 blockade in Treg cells result in enhanced antibacterial immunity in patients with COPD. These studies help to explain the reduced host immunity to bacterial infections that is common in patients with COPD and suggest that Treg cells act as an immune checkpoint in COPD. More importantly, the studies highlight the notion that, in COPD, a paradox exists between pro-inflammatory responses that eradicate pathogens but destroy lung tissue and the immunosuppressive microenvironment that alleviates pro-inflammatory responses but reduces immunity to infection (103). To restore immune function in COPD, therefore, a fine balance is needed between T cell activation and regulation to minimize the impact of T cell-mediated damage, while permitting immunity against respiratory infections. As a consequence, evaluation of infection risk and even bacterial colonization is necessary in screening COPD patients for immunomodulatory therapy (104).

Another study has shown that Treg cells in mouse model indeed promote pulmonary fibrosis by producing the potent profibrotic cytokines TGF-β and PDGF-B (105). Since peribronchial fibrosis is a prominent feature of small airway remodeling in COPD (106) and there is increasing recognition that emphysema and pulmonary fibrosis often coexist in the same patient (107), the potential harmful effects of Treg cells should be taken into account in the pathogenesis of COPD. Although these results are of potential interest, they are preliminary and definitely deserve further investigation.

## Conclusions

In summary, current data suggest that Treg cells have two functions in COPD. One is the beneficial maintenance of peripheral immune tolerance and the other is a deleterious reduction in antibacterial immunity, with a potential profibrotic effect. Because of the methodological complexity in defining Treg cells and the use of different sampling sites, it is not surprising that contrasting results occur in this field. Additional studies are needed to understand the involvement of Treg cells in the different phenotypes or stages of COPD before they can be considered a valid therapeutic target.

## Author Contributions

JH was responsible for analysis of data and manuscript preparation. YS contributed with scientific expertise and manuscript preparation. All authors read and approved the final manuscript.

## Conflict of Interest

The authors declare that the research was conducted in the absence of any commercial or financial relationships that could be construed as a potential conflict of interest.

## Abbreviations

COPD: Chronic obstructive pulmonary disease
tTregs: thymus-derived Tregs
GARP: Glycoprotein A repetitions predominant
rTreg: resting Treg
aTreg cells: activated Treg cells
iTreg: induced Treg cells
TGF-β: transforming growth factor-β
TIGIT: T-cell immunoglobulin and ITIM domains
IPF: idiopathic pulmonary fibrosis
MDSC: myeloid-derived suppressor cells
BMI: Body Mass Index.
